# *Salmonella* Typhi Haplotype 58 biofilm formation and genetic variation in isolates from typhoid fever patients with gallstones in an endemic setting in Kenya

**DOI:** 10.3389/fcimb.2024.1468866

**Published:** 2024-11-13

**Authors:** Peter Muturi, Peter Wachira, Maina Wagacha, Cecilia Mbae, Susan M. Kavai, Michael M. Mugo, Musa Mohamed, Juan F. González, Samuel Kariuki, John S. Gunn

**Affiliations:** ^1^ Centre for Microbiology Research, Kenya Medical Research Institute, Nairobi, Kenya; ^2^ Department of Biology, University of Nairobi, Nairobi, Kenya; ^3^ Department of Medical Services, Ministry of Health, Nairobi, Kenya; ^4^ Center for Microbial Pathogenesis, Abigail Wexner Research Institute at Nationwide Children’s Hospital, Columbus, OH, United States; ^5^ Eastern Africa Office, Drugs for Neglected Diseases initiative, Nairobi, Kenya; ^6^ Infectious Diseases Institute, The Ohio State University, Columbus, OH, United States

**Keywords:** *Salmonella* Typhi, AMR genes, genetic variation, biofilm, typhoid carriage

## Abstract

Although typhoid fever has largely been eliminated in high-income countries, it remains a major global public health concern especially among low- and middle-income countries. The causative agent, *Salmonella enterica* serovar Typhi (*S.* Typhi), is a human restricted pathogen with a limited capacity to replicate outside the human host. Human carriers, 90% of whom have gallstones in their gallbladder, continue to shed the pathogen for an ill-defined period of time after treatment. The genetic mechanisms involved in establishing the carrier state are poorly understood, but *S*. Typhi is thought to undergo specific genetic changes within the gallbladder as an adaptive mechanism. In the current study, we aimed to identify the genetic differences in longitudinal clinical *S*. Typhi isolates from asymptomatic carriers with gallstones in a typhoid endemic setting in Nairobi, Kenya. Whole-genome sequences were analyzed from 22 *S*. Typhi isolates, 20 from stool samples, and 2 from blood samples, all genotype 4.3.1 (H58). Out of this, 19 strains were from four patients also diagnosed with gallstones, of whom three had typhoid symptoms and continued to shed *S*. Typhi after treatment. All isolates had point mutations in the quinolone resistance-determining region (QRDR), and only sub-lineage 4.3.1.2.EA3 encoded multidrug resistance genes. There was no variation in antimicrobial resistance patterns among strains from the same patient/household. Non-multidrug resistant (MDR) isolates formed significantly stronger biofilms *in vitro* than the MDR isolates, p<0.001. A point mutation within the *treB* gene (*treB* A383T) was observed in strains isolated after clinical resolution from patients living in 75% of the households. For missense mutations in Vi capsular polysaccharide genes, *tviE* P263S was also observed in 18% of the isolates. This study provides insights into the role of typhoid carriage, biofilm formation, AMR genes, and genetic variations in *S.* Typhi during asymptomatic carriage.

## 1 Introduction

Typhoid fever (typhoid), a life-threatening systemic infection caused predominantly by *Salmonella enterica* subspecies *enterica* serotype Typhi (*S*. Typhi), remains a common infection and a public health concern in resource poor-settings in parts of sub-Saharan Africa and Asia (Kariuki et al., 2021; Kim et al., 2022; Stanaway et al., 2019). An estimated 9 million new typhoid fever cases occur each year, of which 2%–3% results in death even with adequate antibiotics therapy (Pieters et al., 2018; WHO, 2023). Typical symptoms manifest between 1 and 3 weeks postinfection and encompass elevated prolonged fever, headache, malaise, abdominal pain, diarrhea, constipation, hypersplenism, and rose-colored spots on the chest (World Health Organization, 2019). *S*. Typhi is transmitted via the fecal–oral route in settings with poor standards of sanitation, low levels of hygiene, and inadequate water supply (Crump, 2019; Parry et al., 2010). In addition to inadequate resources, typhoid endemic settings lack a quality public health infrastructure (Dougan and Baker, 2014).

Upon ingestion of contaminated food or water, *S.* Typhi bacteria that survive the hostile gastric acid-rich environment in the stomach are able to replicate in the new host (Ahirwar et al., 2014; Dougan and Baker, 2014). The typhoid bacilli can invade the intestinal mucosa, typically through microfold (M) cells, and establish an initially clinically undetectable infection involving significant systemic dissemination and a transient primary bacteremia (Dougan and Baker, 2014). *S*. Typhi also reach the gallbladder hematogenously during primary bacteremia or shortly thereafter through infected hepatic bile entering the gallbladder (Gaines et al., 1968; Hoffman et al., 2023). *S*. Typhi bacteria can survive, replicate, and evade immune surveillance intracellularly within a modified phagosome known as *Salmonella*-containing vacuole (SCV) (Dougan et al., 2011; Steele-Mortimer, 2008).

Although the majority of patients recover from typhoid fever after an appropriate treatment, some individuals become asymptomatic carriers and shed the infectious typhoid bacilli intermittently in their feces for an ill-defined period of time after apparent clinical resolution. Since the early twentieth century, asymptomatic carriage has been demonstrated to be a source of transmission of typhoid fever, including in the famous case of Mary Mallon (Marineli et al., 2013). Generally, ~2%–5% of acute typhoid cases fail to clear the infection fully within 1 year and develop asymptomatic chronic carriage (Dongol et al., 2012; Gunn et al., 2014; Parry et al., 2002). Approximately 90% of typhoid chronic carriers have gallstones in their gallbladder (Crawford et al., 2010; Lovane et al., 2016).

Persistent colonization of the gallbladder by *S*. Typhi is facilitated by formation of biofilms on the surface of cholesterol gallstones (Crawford et al., 2010; Gonzalez-Escobedo et al., 2011). Biofilms are organized three-dimensional multicellular communities encased in self-produced extracellular polymeric substances (EPS) composed of polysaccharides, extracellular DNA [eDNA], proteins, and lipids (Flemming and Wingender, 2010). For *S*. Typhi to form biofilms on human cholesterol-coated gallstones, a bile-induced EPS and cell-to-cell interaction is required (Crawford et al., 2008). *Salmonella*-species biofilms have several identified EPS components including cellulose, colanic acid, the Vi antigen, curli fimbriae, the O antigen capsule, and some biofilm-associated proteins (Gonzalez-Escobedo et al., 2011; Gibson et al., 2006; Jonas et al., 2007). Formation of biofilms provides several advantages to the bacteria, including enhanced resistance to antibiotics and the host’s immune response, as well as a stable environment that supports long-term colonization (Steenackers et al., 2012). Biofilms account for 80% of chronic infections in humans, leading to increased rates of hospitalization, high healthcare costs, and increased mortality and morbidity rates (Römling and Balsalobre, 2012). Chronic *S*. Typhi colonization usually cannot be resolved with antibiotics; gallbladder resection is the only option, although not always effective (Gonzalez-Escobedo et al., 2011). Biofilm formation leads to continuous shedding and reattachment of planktonic cells, followed by bacteria diffusion in urine and feces (Crawford et al., 2010; Gonzalez-Escobedo and Gunn, 2013). Since *S*. Typhi is a human-restricted pathogen, gallbladder colonization and fecal shedding form a central dogma for further transmission and persistence of typhoid fever.

In the gallbladder, *S*. Typhi is exposed to bile, a complex digestive secretion composed of bile acids, bilirubin, phospholipids, and cholesterol that exhibit strong antimicrobial properties (An et al., 2022; Staley et al., 2017). The molecular mechanisms involved in establishing the carrier state are poorly understood; however, *S*. Typhi is thought to undergo genetic changes within the gallbladder as an adaptive mechanism (Gonzalez-Escobedo et al., 2011; Neiger et al., 2019; Thanh Duy et al., 2020).

Although it is widely accepted that *S*. Typhi carriers contribute to typhoid transmission in endemic settings, little progress has been made in understanding the typhoid carrier state. The current study aimed at identifying the genetic differences in longitudinal clinical *S*. Typhi isolates from carriers, in a typhoid endemic setting in Nairobi, Kenya.

## 2 Methods

### 2.1 Source of bacterial strains

*S.* Typhi strains were isolated from the blood and stool samples of six patients residing in four different households in Mukuru, an informal settlement in Nairobi, Kenya, known for being typhoid-endemic. A typhoid fever patient, along with at least one household contact, provided stool samples for the detection of *S*. Typhi shedding (**Supplementary Table S1**). The laboratory methods for isolation and identification of the isolates are described in our previous publication (Muturi et al., 2024). Briefly, blood samples were inoculated into BACTEC™ culture vials and incubated in a BACTEC blood culture system (Becton, Dickinson and Company, New Jersey, USA) before subculturing on XLD agar. Stool samples were enriched in selenite fecal (SF) broth prior to culturing on XLD agar. *S.* Typhi culture-positive samples were identified using biochemical tests with the Analytical Profile Index 20E (API 20E) system and confirmed by polymerase chain reaction (PCR) amplification of a 1,278-bp fragment of the VI region of the flagellin gene, using primers *tvi*B-F (5′-TCAGCGACTTCTGTTCTATTCAAGTAAGAAAGGGGTACGG-3′) and *tvi*B-R (5′-GCTCCTCACTGACGGACGTGCGAACGTCGTCTAGATTATG-3′). A total of 22 isolates were collected, comprising 2 from blood samples and 20 from stool samples. Nineteen of the isolates (19/22) came from four patients diagnosed with cholelithiasis. Of these patients, three exhibited typhoid symptoms and continued shedding *S.* Typhi post-treatment. Gallstones were confirmed via ultrasound, a standard imaging procedure for assessing gallbladder diseases. One patient with gallstones was asymptomatic but continued to shed *S.* Typhi and lived in the same household (household B) as an acute typhoid fever case who did not have gallstones. In household A, one contact without cholelithiasis shed *S.* Typhi once, whereas the index case, who had gallstones, continued shedding *S.* Typhi after antibiotic treatment. The index case refers to the first identified individual infected with *S.* Typhi and presenting typhoid fever symptoms in each household.

### 2.2 Whole-genome sequencing

DNA extracted from *S*. Typhi strains using GenElute™ Bacterial Genomic DNA Kit (Sigma-Aldrich, Missouri, United States) was prepared for whole-genome sequencing by SeqCoast Genomics (Portsmouth, New Hampshire, United States) using an Illumina DNA Prep Tagmentation kit and unique dual indexes. Sequencing was performed on the Illumina NextSeq 2000 platform using a 300-cycle flow cell kit to produce 2 × 150-bp paired reads as previously described (Grant et al., 2023). PhiX control, 1%–2%, was spiked into the run to support optimal base calling. Read demultiplexing, read trimming, and run analytics were performed using DRAGEN v3.10.12, an on-board analysis software on the NextSeq 2000.

### 2.3 Genome assembly and annotation

Quality-trimming of the reads was done using Trimmomatic (version 0.39) (Bolger et al., 2014). Read error was corrected using SPAdes (v. v3.13.1) (Bankevich et al., 2012). The reads were assembled into contigs using SPAdes (wrapped in Unicycler). Read mapping was done using Bowtie2 and SAMtools (wrapped in Unicycler, version 0.4.4) (Langmead and Salzberg, 2012; Li et al., 2009; Wick et al., 2017). Pilon (v1.24) (wrapped in Unicycler) was used in polishing of each assembly (Walker et al., 2014). Gene prediction and functional annotation were performed using BAKTA (version 1.5.1) (Schwengers et al., 2021). The annotation pipeline was as follows: prediction of protein-coding genes using Prodigal, tRNA identification using tRNAscan-SE (Chan and Lowe, 2019), tRNA and tmRNA identification using Aragorn (Laslett and Canback, 2004), prediction of rRNA sequences using Infernal and the Rfam database (Nawrocki and Eddy, 2013), CRISPR prediction using PILER-CR (Edgar, 2007), antimicrobial resistance gene identification using AMRFinderPlus (Feldgarden et al., 2021), prediction of signal peptides using DeepSig (Savojardo et al., 2018), prediction of transposases using ISFinder (Siguier et al., 2006), and computation of codon usage biases for each amino acid using the codonUsage.py script (https://github.com/Arkadiy-Garber/BagOfTricks) (Garber, 2024).

### 2.4 Genotype identification and bacteria clustering

Identification and clustering of *S*. Typhi genotypes was performed using Pathogenwatch (https://pathogen.watch/), a web-based platform with several different components (Argimón et al., 2021; Wong et al., 2016). The platform provides compatibility with *S*. Typhi typing information for MLST (Sharma et al., 2016), *in silico* serotyping (SISTR) (Yoshida et al., 2016), and an SNP genotyping scheme (GenoTyphi) (Wong et al., 2016). *S.* Typhi assemblies (in fasta format) were uploaded to the platform (https://pathogen.watch/upload/fasta) for the analysis. A collection of uploaded sequences was created in Pathogenwatch to generate a phylogenetic tree. *S*. Typhi genotype 2.1 (GenBank Accession: SAMEA2158302) was used as the outgroup. The phylogenetic tree generated by Pathogenwatch (Newick format) was downloaded and exported to Microreact (https://microreact.org) for visualization. Associated metadata in CSV format was also exported to Microreact, and a phylogenetic tree in Newick format, reflecting branch lengths corresponding to SNP/genetic distances, was obtained.

### 2.5 Antimicrobial susceptibility testing

Antimicrobial susceptibility testing was performed using the disk diffusion technique (Reller et al., 2009) for all antimicrobials commonly used in Kenya for typhoid fever treatment including ampicillin (10 µg), tetracycline (30 µg), co-trimoxazole (25 µg), chloramphenicol (30 µg), amoxicillin–clavulanate (20/10 μg), cefpodoxime (30 µg), ceftazidime (30 µg), ceftriaxone (30 µg), cefotaxime (30 µg), azithromycin (15 μg), ciprofloxacin (5 µg), nalidixic acid (10 µg), kanamycin (30 μg), and gentamicin (10 μg). The diameter of the zone of inhibition was measured after 18 h–24 h, and results were interpreted according the Clinical and Laboratory Standards Institute (CLSI), guidelines for *Salmonella* (Clinical and Laboratory StandardsInstitute, 2023).

### 2.6 Screening of antimicrobial resistance genes

NCBI Antimicrobial Resistance Gene Finder Plus (AMRFinderPlus, v3.12.8) (https://github.com/ncbi/amr/wiki) was used to identify acquired antimicrobial resistance genes and known resistance-associated point mutations in *S*. Typhi-assembled nucleotide sequences (Feldgarden et al., 2021).

### 2.7 Variant calling

To identify genetic variations in the longitudinal clinical isolates relative to acute isolates (*S*. Typhi isolated during typhoid fever diagnoses), *S*. Typhi strains isolated before each typhoid index case was treated with antibiotics were used as the reference genome and were compared with those isolated after treatment (follow-up isolates), and/or those isolated from household contacts using breseq v0.38.2 (https://github.com/barricklab/breseq), a computational pipeline for finding mutations relative to a reference sequence in short-read DNA (Deatherage and Barrick, 2014). The variant calling pipeline was as follows: quality-filtering of raw reads using Trimmomatic v0.39 (Bolger et al., 2014), mapping of reads against a reference genome (first strain isolated from the index case), analysis of possible mutations based on mapping data, identification of mutations, and tabular summaries of mutation profile across samples (Langmead and Salzberg, 2012).

### 2.8 Plasmid identification

To identify plasmids carrying acquired AMR genes in the isolated *S.* Typhi strains, a plasmid detection tool PLASMe (v1.1) was used (https://github.com/HubertTang/PLASMe). The tool uses the alignment component in PLASMe to identify closely related plasmids whereas diverged plasmids are predicted using order-specific Transformer models (Tang et al., 2023). Assembled *S*. Typhi genomes were used for this analysis.

### 2.9 *In vitro* biofilm formation assays

Because of the importance of biofilm formation on gallstones in chronic carriage (Crawford et al., 2010; Gunn et al., 2014), the biofilm-forming ability of all the 22 human *S*. Typhi isolates was tested under gallbladder simulating conditions as previously described (González et al., 2018). Briefly, *S*. Typhi biofilms were grown on non-treated polystyrene 96-well plates (Corning, Kennebunkport, ME). To simulate growth conditions on gallstones, wells in two plates were precoated with cholesterol by adding a solution of 5 mg/mL in 1:1 isopropanol:ethanol and air-dried overnight (Crawford et al., 2008). A pure colony of *S*. Typhi on an XLD agar plate was cultured in Tryptone Soy Broth (TSB). Overnight (O/N) cultures in broth were normalized to OD_600_ = 0.8 and diluted 1:2,500 in TSB or TSB containing 2.5% human bile, and 100 µL/well was dispensed into the plates. The plates were incubated at 25°C in a Fisherbrand™ nutating mixer (Thermo Fisher Scientific; Hampton, NH) at 24 rpm for 96 h. Media (TSB or TSB containing bile) were changed after every 24 h for consistent *S*. Typhi biofilm growth. Plates were emptied on the fourth day and washed twice before heat fixing at 60°C for 1 h. The biofilms were stained using a crystal violet solution and acetic acid (33%) used to elute crystal violet before reading the OD_570_. GraphPad Prism 9.5 was used to analyze the biofilm formation results. One-way analysis of variance (ANOVA) was used to test the level of significance in biofilm formation between the different *S*. Typhi sub-lineages and in different conditions, i.e., biofilms in absence of cholesterol and bile, in cholesterol-coated plates in absence of bile, and in presence of cholesterol and bile. Student’s t-test was used to test the level of significance in biofilm formation in strains isolated before treatment vs. last strains shed by the patient, P-values less than 0.05 (P<0.05) were considered significant.

### 2.10 Ethical statement

#### 2.10.1 Ethical approval

This study was conducted in accordance with the ethical standards and guidelines of Scientific and Research Unit (Approval No. SERU4227) of Kenya Medical Research Institute (KEMRI).

#### 2.10.2 Informed consent

Informed consent was obtained from all participants involved in the study.

#### 2.10.3 Laboratory protocols

The biofilm samples were handled following strict laboratory protocols to ensure participant safety and environmental compliance.

## 3 Results

### 3.1 Genotype identification and clustering tree

**Figure 1:**
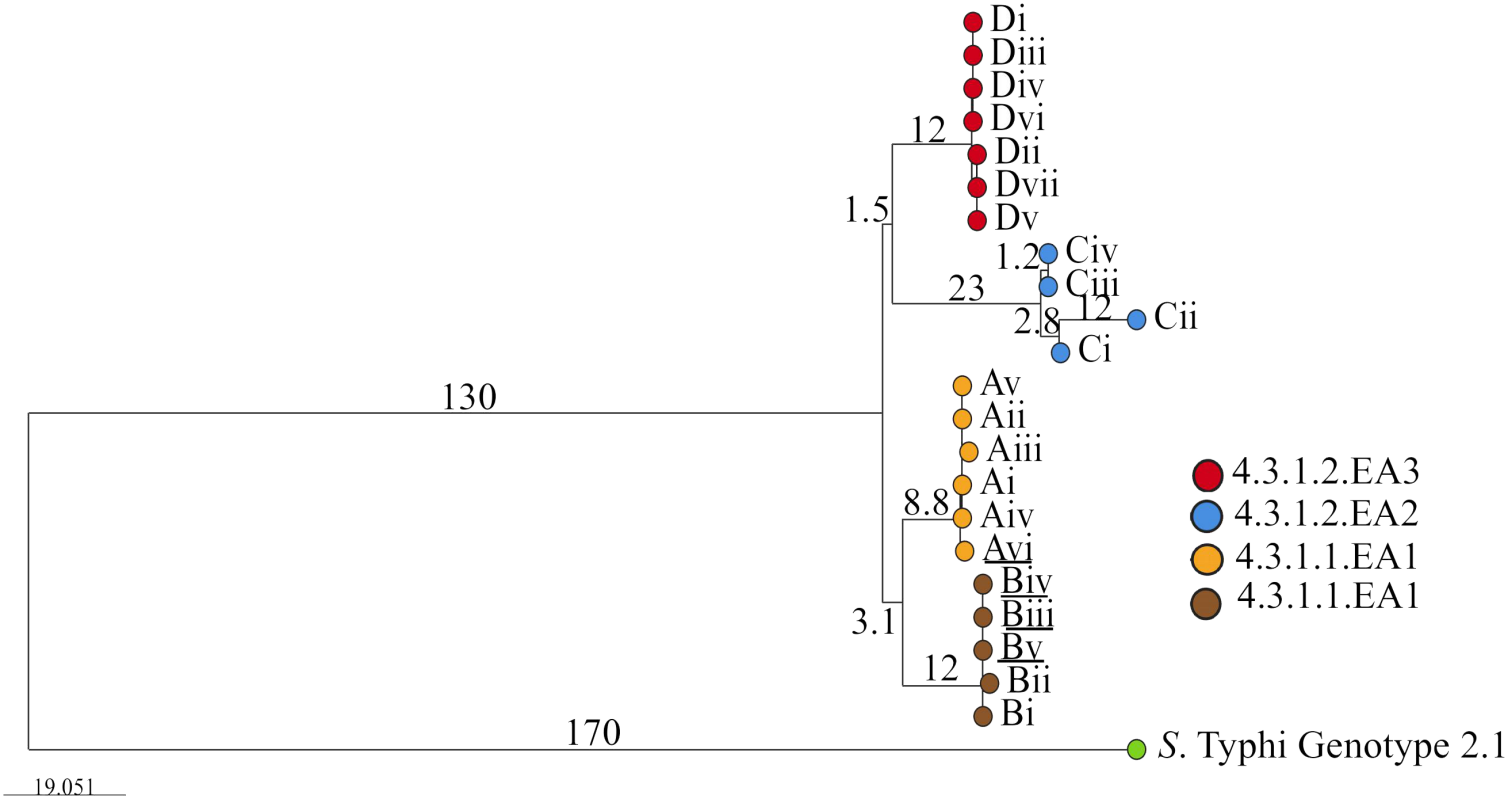
Phylogenetic tree showing the evolutionary relationship between *S*. Typhi isolated from the study participants living in the different households. Roman numbers indicate specific isolates from the different households (A, B, C and D). Branch tips are color-coded according to the household of isolation. Underlined are isolates from household contacts (Avi, Biii, Biv, and Bv). The bar represents branch length scale bar, indicating evolutionary distance. Branch length corresponds to SNP/genetic distances. The tree can be visualized here https://microreact.org/project/qkEmefYaRCmJRpBKgJnzDS-s-typhi-isolated-from-typhoid-acute-cases-and-asymptomatic-carriers-in-kenya.

Given the highly structured nature of the *S*. Typhi population, with numerous subclades associated with specific geographical regions and antimicrobial resistance patterns, the genotypes responsible for asymptomatic carriage in the current study were identified using a combination of three methods employed by Pathogenwatch for *S*. Typhi typing: multi-locus sequence typing (MLST), *in silico* serotyping (SISTR), and the SNP genotyping scheme (GenoTyphi). Among the 22 bacterial isolates, which include two from blood samples and 20 from stool samples collected at various time points from typhoid fever patients and asymptomatic carriers of *S*. Typhi (**Table 1**), all were identified as genotype 4.3.1 (*S*. Typhi Haplotype 58 [H58]). Of these, 11 isolates from households A and B were classified under lineage 4.3.1.1, whereas the remaining 11 isolates from households C and D fell under lineage 4.3.1.2. (**Figure 1**). Lineage 4.3.1.2 was further divided into two sub-lineages: 4.3.1.2.EA2 (four isolates from the index case in household C) and 4.3.1.2.EA3 (seven isolates from the index case in household D). Each patient shed *S*. Typhi belonging to only one lineage or sub-lineage. From household A, isolates (i)–(v) were from the index case whereas isolate (vi) was from a household contact. Isolates (i) and (ii), household B, were from the index case whereas (iii), (iv), and (v) were from an asymptomatic household contact living with the index case. All household C isolates belonged to sub-lineage 4.3.1.2.EA2 whereas all household D isolates belonged to sub-lineage 4.3.1.2.EA3. Longitudinal *S*. Typhi isolates were collected at different time points as shown in **Table 1**.

**Table 1:** Time of isolation/shedding of *S*. Typhi.

Household	Category of study participant	Time of isolation of *S*. Typhi isolates (no. of days after the index case was diagnosed with typhoid fever)						
		(i)	(ii)	(iii)	(iv)	(v)	(Vi)	(vii)
A	Index case*	0	31	35	39	88	–	–
	Household contact	–	–	–	–	–	31	–
B	Index case	0	0	–	–	–	–	–
	Household * contact	–	–	29	33	40	–	–
C	Index case*	0	22	78	170	–	–	–
D	Index case*	0	0	21	28	35	76	107

Roman numerals indicate the order of isolation of *S*. Typhi strains from each household.

*Patients with gallstones in their gallbladder. Index cases had symptoms at the time of recruitment, whereas household contacts were asymptomatic and living with a typhoid fever acute case.

Day 0 isolates (Bi and Di from blood samples, and Ai, Bii, Ci, and Dii from stool samples) were collected at the time of diagnosis from patients with typhoid fever.

Index case is the first identified case infected with *S.* Typhi in each of the four households.

### 3.2 Antimicrobial resistance genes

Different antimicrobial resistance patterns were observed in the isolated *S*. Typhi strains. The seven isolates belonging to sub-lineage 4.3.1.2.EA3 (from household D) were multidrug resistant, all expressing the following acquired antimicrobial resistance genes: *sul1*, *dfrA7*, *catA1*, *aph(6)-Id*, *aph(3”)-Ib*, *sul2*, and *bla_TEM-1_*, and a point mutation in the Quinolone Resistance Determining Region (QRDR) of *gyrA* (*gyrA* S83Y). Phenotypic susceptibility data showed that these seven isolates were resistant to ampicillin, chloramphenicol, trimethoprim–sulfamethoxazole, and nalidixic acid but non-susceptible to ciprofloxacin. The four sub-lineage 4.3.1.2.EA2 isolates (*S*. Typhi strains from Household C) had a *gyrB* S464F mutation in the QRDR, and all were non-susceptible to nalidixic acid and ciprofloxacin according to phenotypic susceptibility results. The third group, lineage 4.3.1.1, had six strains (household A isolates) with a *gyrB* S464F mutation, also demonstrating non-susceptibility to nalidixic acid and ciprofloxacin. The other five lineage 4.3.1.1 strains (household B isolates) had *gyrA* S83F mutations in the QRDR and showed resistance to nalidixic acid and non-susceptibility to ciprofloxacin (**Table 2**). *S*. Typhi isolates carrying genes that confer resistance to ampicillin, sulfamethoxazole–trimethoprim, and chloramphenicol were classified as multidrug resistant (MDR), whereas those lacking these genes were classified as non-MDR.

**Table 2:** Antibiotic resistance profiles in isolated *S.* Typhi.

Antimicrobial resistance (AMR)			*Salmonella* Typhi isolates N (%)			
Resistance pattern	AMR genes	Antibiotics	Household A (4.3.1.1.EA1)	Household B (4.3.1.1.EA1)	Household C (4.3.1.2.EA2)	Household D (4.3.1.2.EA3)
MDR	*sul1*, *dfrA7*, *catA1*, *aph(6)-Id*, *aph(3’’)-Ib*, *sul2*, *bla_TEM-1_* and point mutation on DNA gyrase sub-unit A (*gyrA* S83Y)	Resistant to AMP, SXT, CHL and NAL, and reduced susceptibility to CIP	**-**	**-**	**-**	7/7 (100%)
Non-MDR	Point mutation on DNA gyrase sub-unit A (*gyrA* S83F)	Resistant to NAL and reduced susceptibility to CIP	**-**	5/5 (100%)	**-**	**-**
	Point mutation on DNA gyrase sub-unit B (*gyrB* S464F)	Reduced susceptibility to NAL and CIP	6/6 (100%)	**-**	4/4 (100%)	**-**

MDR, multidrug resistant.

AMP, ampicillin, SXT, sulfamethoxazole–trimethoprim, CHL, chloramphenicol, NAL, nalidixic acid, CIP, ciprofloxacin.

*sul1* and *sul2* mediate resistance to sulfonamide, *bla_TEM-1_* encodes broad-spectrum β-lactamase, *aph(6)-Id* and *aph(3”)-Ib* confer resistance to streptomycin, *dfrA7* confers resistance to trimethoprim, and *catA1* confers resistance to chloramphenicol.

### 3.3 Variant calling

Comparative genome sequencing of the *S*. Typhi isolates revealed several key mutations across different sub-lineages. In the sub-lineage 4.3.1.2.EA3 isolates from household D, a missense mutation in the *treB* gene (*treB* A383T) was identified in three out of seven isolates. Additionally, a second missense mutation in the *tviE* gene (*tviE* P263S), which codes for the Vi polysaccharide biosynthesis protein TviE, was observed in four out of seven isolates. Notably, two of these isolates carried both mutations. A nonsense mutation (E237*) was also found in 1/7 of the isolates, affecting a locus coding for an integrase/transposase family protein similar to DDE-type integrase/transposase/recombinase in *S.* Typhi CT18 (GenBank: MEM6073260.1). The first follow-up isolate from household C (sub-lineage 4.3.1.2.EA2, isolate Cii) exhibited a total of 16 mutations. This included seven silent mutations, eight missense mutations, and a deletion in the *mutL* gene, which encodes the DNA mismatch repair endonuclease MutL (**Table 3** and **Supplementary Table S2** and **Supplementary File 3**). The second and third follow-up isolates from the same patient (Ciii and Civ) showed two silent mutations, one in the *yccC* gene, coding for a putative membrane protein, and another in the *tehA* gene, coding for a dicarboxylate transporter/tellurite-resistance protein (*tehA* A124A and *yccC* R199R mutations respectively). These isolates also had missense mutations in the *amiA* gene, which encodes N-acetylmuramoyl-L-alanine amidase (*amiA* V145A), and an additional mutation in the *hpaX* gene (*hpaX* K124E), encoding 4-hydroxyphenylacetate permease. Notably, the second follow-up isolate (Ciii) had an additional missense mutation (D78G) in a locus coding for phage baseplate assembly protein V (GenBank: MEM6147547.1). In the follow-up isolates from households A and B, which belong to sub-lineage 4.3.1.1, several mutations were detected. The first follow-up *S*. Typhi isolate from the index case in household A showed no mutations. However, the second follow-up sample (Aiii) had a single nucleotide polymorphism in the *crl* gene (*crl* L38P), which encodes a sigma factor-binding protein. The third and fourth follow-up isolates from the same patient (Aiv and Av) each had a silent mutation in the *tnpA* gene (*tnpA* Y41Y), which codes for IS200/IS605 family transposase. Additionally, an isolate from a household contact in the same household (Avi) had a missense mutation in the *treB* gene (*treB* A383T). From household B, *S*. Typhi isolated from the stool of the index case before treatment (isolate Bii) had a missense mutation in the *waaK* gene (*waaK* P167L), which encodes lipopolysaccharide N-acetylglucosaminyltransferase. This mutation was not present in the strain isolated from the patient’s blood sample collected on the same day.

**Table 3:** Missense mutations in *S.* Typhi strains isolated from index cases after apparent clinical resolution and from asymptomatic household contacts.

Gene/locus (GenBank accession)	Seq change	Household A 4.3.1.1.EA1					Household B 4.3.1.1.EA1				Household C 4.3.1.2.EA2			Household D 4.3.1.2.EA3					
		ii	iii	iv	v	vi	ii	>iii	iv	v	ii	iii	iv	ii	iii	iv	v	vi	vii
*crl*	L38P		✓																
*treB*	A383T					✓					✓			✓			✓		✓
*waaK*	P167L						✓												
MEM6073260.1	E237*														✓				
*tviE*	P263S															✓	✓	✓	✓
MEM6145904.1	M224V										✓								
*yhhY*	R73H										✓								
MEM6148091.1	R51W										✓								
*yihT*	R270C										✓								
*rfbP*	D196G										✓								
*galR*	Y98H										✓								
*dnaE*	E953G										✓								
*hpaX*	K124E											✓	✓						
*amiA*	V145A											✓	✓						
MEM6147547.1	D78G											✓							

Roman numbers in red represent isolates from household contacts.

Follow-up isolates from index cases are in bold and underlined.

✓ indicates isolates with observed missense mutations.

These color codes visually differentiate the isolates from each household.

### 3.4 Plasmid identification

*S*. Typhi genotype 4.3.1 lineages/sub-lineages identified in this study were found to contain plasmids. Genes conferring multidrug resistance in *S*. Typhi were detected in two different plasmids in sub-group 4.3.1.2.EA3 (household D) strains. The antimicrobial resistance genes *sul1*, *dfrA, catA1, aph(6)-Id, aph(3”)-Ib, and sul2* were detected in a plasmid with sequences corresponding to *S*. Typhi strain 311189_252186 plasmid pHCM1, whereas *bla_TEM-1_* was detected in a plasmid with sequences corresponding to *E. coli* NES6 plasmid pN-ES-6-1 (**Table 4**). The two plasmids were not detected in non-MDR *S*. Typhi Isolates.

**Table 4:** Contigs carrying AMR genes in household D *S*. Typhi isolates and plasmids with similar sequences from the NCBI public database.

AMR genes	Isolate	Contig containing AMR genes (GenBank Accession)	Contig length	GenBank Accession number of plasmids with sequences similar to contigs carrying AMR genes	Inc type	Score
*sul1*, *dfrA7*, and *catA1*	Di	JBCHBR010000039.1	8,971	NZ_CP029895.1 (plasmid pHCM1)	IncH1	0.99
	Dii	JBCHBQ010000041.1	8,971			0.99
	Diii	JBCHBP010000040.1	8,971			0.99
	Div	JBCHBO010000040.1	8,971			0.99
	Dv	JBCHBN010000041.1	8,971			0.99
	Dvi	JBCHBM010000041.1	8,971			0.99
	Dvii	JBCHBL010000041.1	8,971			0.99
*aph(6)-Id*, *aph(3”)-Ib* and *sul2*	Di	JBCHBR010000046.1	4,386			0.99
	Dii	JBCHBQ010000048.1	4,386			0.99
	Diii	JBCHBP010000047.1	4,386			0.99
	Div	JBCHBO010000047.1	4,386			0.99
	Dv	JBCHBN010000048.1	4,386			0.99
	Dvi	JBCHBM010000048.1	4,386			0.99
	Dvii	JBCHBL010000048.1	4,386			0.99
*blaTEM-1*	Di	JBCHBR010000052.1	1,831	LC553463.1 (plasmid pN-ES-6-1)	IncF1	0.99
	Dii	JBCHBQ010000055.1	1,831			0.99
	Diii	JBCHBP010000053.1	1,831			0.99
	Div	JBCHBO010000053.1	1,831			0.99
	Dv	JBCHBN010000055.1	1,831			0.99
	Dvi	JBCHBM010000054.1	1,831			0.99
	Dvii	JBCHBL010000055.1	1,831			0.99

### 3.5 Biofilm formation

**Figure 2:**
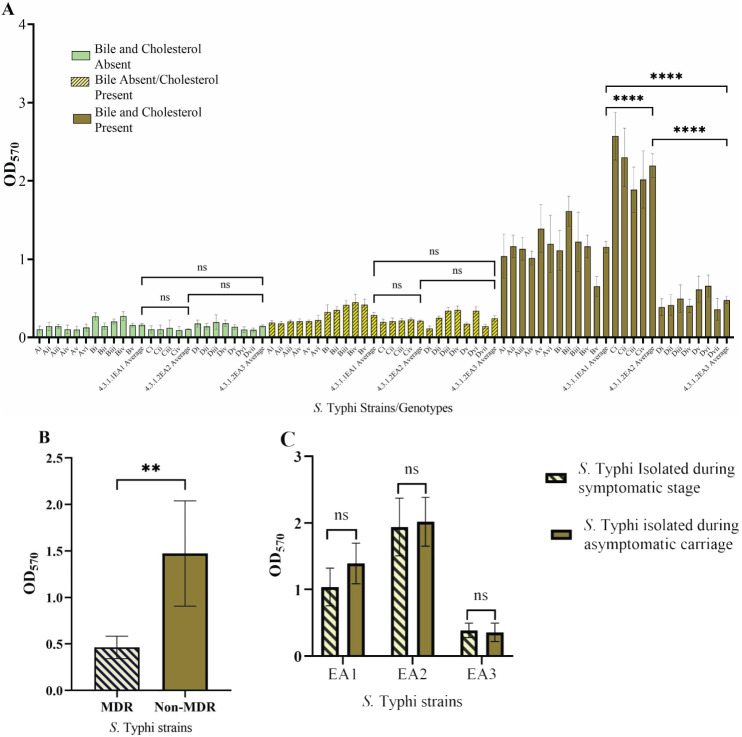
*Salmonella* Typhi biofilms. **(A)** Quantity of biofilms after growth in presence/absence of cholesterol and/or bile. **(B)** Biofilm formation by MDR *S*. Typhi strains vs. non-MDR strains. **(C)** Comparison of biofilms formed by *S*. Typhi strains isolated before treatment vs. after treatment. Error bars represent SEM, ****P<0.001; **P<0.05; ^ns^ *P*≥0.5.

We wanted to determine if biofilm-forming ability is correlated with the stage of typhoid fever/carriage, antimicrobial resistance, or the presence of plasmids. Biofilms were examined in gallbladder-simulating conditions. There was varying ability to form biofilms under *in vitro* conditions in the *S.* Typhi strains tested. All isolates formed weak biofilms in absence of both cholesterol and bile (OD_570_ below 0.3) and significantly strong biofilms in the presence of cholesterol and 2.5% human bile (**Figure 2A**). Differences in biofilm forming ability were observed across the identified genotype 4.3.1 lineages/sub-lineages. The sub-lineage 4.3.1.2.EA2 formed the strongest biofilms (OD_570_ slightly above 2.0), whereas sub-lineage 4.3.1.2.EA3 formed relatively weak biofilms (OD_570_ below 0.5) even in the presence of cholesterol and human bile (**Figure 2A**). However, there was no statistical significance in biofilm forming ability in strains isolated during the symptomatic vs. asymptomatic stage in each of the three sub-groups of *S.* Typhi (**Figure 2C**). The weak biofilm-forming isolates from sub-lineage 4.3.1.2.EA3 had acquired antimicrobial resistance (AMR) genes encoded on plasmids. Sub-lineages 4.3.1.2.EA2 and 4.3.1.1.EA1 did not have identifiable acquired AMR genes, but isolates in these subgroups formed significantly stronger biofilms in the presence of both cholesterol and human bile (**Figure 2B**). Strains from symptomatic patients and asymptomatic carriers exhibited no statistically significant difference in biofilm-forming ability across the three *S*. Typhi subgroups (**Figure 2C**).

## 4 Discussion

Although typhoid fever has largely been eliminated in high-income countries, it remains a major global public health concern especially among low- and middle-income countries (Kim et al., 2022). Haplotype 58 (H58), which is associated with antimicrobial resistance, has also been reported from other parts of sub-Saharan Africa and Southeast Asia (Maes et al., 2020; Pragasam et al., 2020). In this study, H58 (genotype 4.3.1) was identified as the single genotype shed by four cholelithiasis patients living in a typhoid endemic setting in Nairobi, Kenya. *S*. Typhi H58 is the most dominant genotype in many parts of Southeast and South Asia as well as in East Africa and has spread globally (Park et al., 2018; Wong et al., 2015). Three H58 east African subgroups (4.3.1.1.EA1, 4.3.1.2.EA2, 4.3.1.2.EA3) previously reported circulating in the current study setting by our group (Kariuki et al., 2021) were the main lineages/sub-lineages shed by the cholelithiasis patients. The most abundant subgroup was 4.3.1.1.EA1 with 11/22 (50%) isolates, originating from individuals living in two different households. In one of these households, an acute case shed an *S.* Typhi belonging to the same sub-group as an asymptomatic household contact who was also diagnosed with gallstones. This suggests possible transmission of the pathogen by the carrier to the household contact (household B). From a different household, a typhoid patient also diagnosed with gallstones continued to shed sub-lineage 4.3.1.2.EA2, whereas in the fourth household, *S.* Typhi sub-lineage 4.3.1.2.EA3 strains were isolated from stool samples collected from an acute case after treatment. Unlike the other two sub-groups, the sub-lineage 4.3.1.2.EA3 strains had MDR genes, showing resistance to ampicillin, sulfamethoxazole–trimethoprim, and chloramphenicol. All 22 *S.* Typhi isolates had point mutations in the QRDR, conferring reduced susceptibility to ciprofloxacin, a drug of choice for treating typhoid fever. There was no variation noted in antimicrobial resistance patterns among strains isolated from the patients in the same household.

Multidrug resistance genes were not detected in 4.3.1.1.EA1 and 4.3.1.2.EA2 *S*. Typhi genomes, but the strains belonging to these subgroups formed significantly stronger biofilms as compared with the MDR sub-lineage 4.3.1.2.EA3 strains. Biofilms act as a physical barrier protecting bacteria from killing by antimicrobials including antibiotics. A previous study demonstrated the role of biofilms in protecting *Salmonella* from ciprofloxacin (González et al., 2018). We hypothesize that the 4.3.1.1.EA1 and 4.3.1.2.EA2 sub-lineages form better biofilms to counteract the absence of antimicrobial resistance factors or, conversely, that lineage 4.3.1.2.EA3 has lost biofilm-related genes because it possesses acquired genes (in plasmids) encoding strong antimicrobial resistance. To the best of our knowledge, this is the first study comparing biofilm forming ability in different *S*. Typhi lineages. The mechanism leading to differences in biofilm formation in isolates from the same genotype will need to be further investigated.

Genetic variations were observed in *S*. Typhi from asymptomatic carriers, with a *treB* A383T point mutation being observed in at least one isolate from each of the four households. The *treB* gene codes for PTS trehalose transporter subunit IIBC. As seen in **Table 3**, household B, some of the mutations observed in the first follow-up isolate were not detected in *S*. Typhi strains isolated during the consecutive follow-ups. However, some mutations were observed in more than one strain isolated from the same patient. From the patient shedding sub-lineage 4.3.1.2.EA3 strains, the *tviE* P263S mutation was observed in the fourth isolate and all strains isolated thereafter. This suggests that some mutations are retained in the population during asymptomatic carriage, whereas others are not. Mutations that confer a selective advantage to *S*. Typhi during carriage are likely maintained, whereas those that negatively impact the pathogen are eliminated. Additionally, changes in environmental conditions during shedding from the gallbladder may alter the selective pressure on mutations, resulting in the retention or loss of specific mutations. Although no strain was isolated directly from gallbladder in our study, mutations in the *tviE* gene were also observed in *S.* Typhi gallbladder genome sequences in a previous study (Thanh Duy et al., 2020). The *tviE* gene facilitates the polymerization and translocation of the Vi capsule (Virlogeux et al., 1995). Vi capsular polysaccharide, an antiphagocytic capsule, covers the surface of *S*. Typhi allowing it to selectively evade phagocytosis by human neutrophils whereas promoting human macrophage phagocytosis (Zhang et al., 2022). This crucial virulence factor in *S*. Typhi (Vi) also plays a key role in the development of vaccines against typhoid fever (Hu et al., 2017). However, additional research will be required to understand if this mutation alters the expression of Vi antigen to benefit *S*. Typhi pathogenesis or chronic carriage. Both nonsense and non-synonymous mutations have been previously reported in gallbladder *S*. Typhi isolates, particularly in genes encoding hypothetical proteins, membrane lipoproteins, transport/binding proteins, surface antigens, and carbohydrate degradation enzymes (Thanh Duy et al., 2020).

The primary limitation of this study is the unavailability of gallbladder isolates from patients shedding *S*. Typhi for comparison with those obtained from stool and blood samples. Additionally, there were no *S*. Typhi isolates from other genotypes available for comparative analysis.

## 5 Conclusions

The resistance patterns in *S*. Typhi did not change during the duration of asymptomatic carriage in study participants, but these individuals continued to shed and transmit drug-resistant strains of this pathogen. This included strains isolated from the patients in the same household, suggesting that asymptomatic typhoid carriers are responsible for the transmission and persistence of drug-resistant *S*. Typhi in the study setting. No specific set of AMR genes was linked to asymptomatic carriage. Mutations in *S*. Typhi were observed to occur during carriage including those in the Vi antigen locus. Sub-lineages analyzed in this study that were not multidrug resistant showed the ability to form stronger biofilms than the multidrug resistant strains. This study provides some insights into mutations, drug resistance, and biofilm formation during typhoid carriage, and this information may be used to influence public health approaches aimed at reducing carriage, persistence, and transmission of *S*. Typhi.

## Data Availability

The data presented in the study are deposited in the NCBI GenBank repository, accession number PRJNA1101423.
