# Supplementary material for: Salmonella Typhi Haplotype 58 biofilm formation and genetic variation in isolates from typhoid fever patients with gallstones in an endemic setting in Kenya
Source: Front Cell Infect Microbiol. 2024 Nov 13;14:1468866. doi: 10.3389/fcimb.2024.1468866 (PMC11599249; doi:10.3389/fcimb.2024.1468866)
Supplement: Supplementary file 1 [file SupplementaryFile1.pdf]

Household A

sigma factor-binding protein Crl

25 A (i)-Ref, GenBank: MEM6204067.1

MTLPSGHPKSRLIKKFTALGPYIREGQCEDNRFFFDCLAVCVNVKPAPEKREFWGWWMELEAQEKRFTYR
YQFGLFDKEGNWTVVPINETEVVERLEYTLREFHEKLRDLLISMELALEPSDDFNDEPVKLSA

33 A (iii), *crL* L38P, GenBank: MEM6102875.1

MTLPSGHPKSRLIKKFTALGPYIREGQCEDNRFFFDCPAVCVNVKPAPEKREFWGWWMELEAQEKRFTYR
YQFGLFDKEGNWTVVPINETEVVERLEYTLREFHEKLRDLLISMELALEPSDDFNDEPVKLSA

PTS trehalose transporter subunit IIBC

25 A (i)-Ref, GenBank: MEM6202349.1

MSKVKQADIDRLIDLVGGRDNIATVSHCITRLRFVLHQPANARPKEIEQLPMVKGCFTNAGQFQVVIGTE
VGDYYNALLETTGKAYADKEQAKKAARQNMKWHEQLISHFAEIFFPLLPALISGGLILGFRNVIGDVPMS
HGQTLAQMHPALKTLYDFLWLIGEAIFFYLPVGICWSAVKKMGGTPILGIVLGVTLVSPQLMNAYLLGQQ
TPDVWNFGVFSIEKIGYQAQVIPALLAGLALGFIETRLKRIVPDYLYLVVVPVCSLILAVFLAHAFIGPF
GRMIGDGVAFAVRYLMTGSFAPIGAALFGFLYAPLVITGVHQTTLAIDMQMVQSMGGTPVWPLIALSNIA
QASAVVGIIISSRKHNEREISVPAAISAYLGVAEPAMYSINIKYRFPMLCAMIGSGLAGLLCGLNGVMAN
GIGVGGLPGILSIQPTYWQVFAMAMVIAIVIPVILTTFIYQRKHRQGTLQIV

31 A (vi), *treB* A383T, GenBank: MEM6142272.1

MSKVKQADIDRLIDLVGGRDNIATVSHCITRLRFVLHQPANARPKEIEQLPMVKGCFTNAGQFQVVIGTE
VGDYYNALLETTGKAYADKEQAKKAARQNMKWHEQLISHFAEIFFPLLPALISGGLILGFRNVIGDVPMS
HGQTLAQMHPALKTLYDFLWLIGEAIFFYLPVGICWSAVKKMGGTPILGIVLGVTLVSPQLMNAYLLGQQ
TPDVWNFGVFSIEKIGYQAQVIPALLAGLALGFIETRLKRIVPDYLYLVVVPVCSLILAVFLAHAFIGPF
GRMIGDGVAFAVRYLMTGSFAPIGAALFGFLYAPLVITGVHQTTLAIDMQMVQSMGGTPVWPLIALSNIA
QASAVVGIIISSRKHNEREISVPAAISAYLGVTEPAMYSINIKYRFPMLCAMIGSGLAGLLCGLNGVMAN
GIGVGGLPGILSIQPTYWQVFAMAMVIAIVIPVILTTFIYQRKHRQGTLQIV

Household B

lipopolysaccharide N-acetylglucosaminyltransferase

84 B (i)-Ref, GenBank: MEM6116494.1

MIKKIIFTVTPIFSIPPRGAAAVETWIYQVAKRLSIPNAIACIKNAGYPEYNKINDNCDIHYIGFSKVYK
RLFQKWARLDPLPYSQRILNIRDKVTTQEDSVIVIHNSMKLYRQIRERNPNAKLVMHMHNAFEPELPDTD
AKIIVPSQFLKAFYEERLPAAAVSIVPNGFCAETYKRNPQDNLRQQLNIAEDATVLLYAGRISPDKGILL
LLQAFKQLRTLRSNIKLVVVGDPYASRKGEKAEYQKKVLDAAKEIGTDCIMAGGQSPDQMHNFYHIADLV
IVPSQVEEAFCMVAVEAMAAGKAVLASKKGGISEFVLDGITGYHLAEPMSSDSIINDINRALADKERHQI
AEKAKSLVFSKYSWENVAQRFEEQMKSWFDK

83 B (ii), *waaK* P167L, GenBank: MEM6092468.1

MIKKIIFTVTPIFSIPPRGAAAVETWIYQVAKRLSIPNAIACIKNAGYPEYNKINDNCDIHYIGFSKVYK
RLFQKWARLDPLPYSQRILNIRDKVTTQEDSVIVIHNSMKLYRQIRERNPNAKLVMHMHNAFEPELPDTD
AKIIVPSQFLKAFYEERLPAAAVSIVLNGFCAETYKRNPQDNLRQQLNIAEDATVLLYAGRISPDKGILL
LLQAFKQLRTLRSNIKLVVVGDPYASRKGEKAEYQKKVLDAAKEIGTDCIMAGGQSPDQMHNFYHIADLV
IVPSQVEEAFCMVAVEAMAAGKAVLASKKGGISEFVLDGITGYHLAEPMSSDSIINDINRALADKERHQI
AEKAKSLVFSKYSWENVAQRFEEQMKSWFDK

Household C

PTS trehalose transporter subunit IIBC

30 C (i)-Ref, GenBank: MEM6080135.1

MSKVKQADIDRLIDLVGGRDNIATVSHCITRLRFVLHQPANARPKEIEQLPMVKGCFTNAGQFQVVIGTE
VGDYYNALLETTGKAYADKEQAKKAARQNMKWHEQLISHFAEIFFPLLPALISGGLILGFRNVIGDVPMS
HGQTLAQMHPALKTLYDFLWLIGEAIFFYLPVGICWSAVKKMGGTPILGIVLGVTLVSPQLMNAYLLGQQ
TPDVWNFGVFSIEKIGYQAQVIPALLAGLALGFIETRLKRIVPDYLYLVVVPVCSLILAVFLAHAFIGPF
GRMIGDGVAFAVRYLMTGSFAPIGAALFGFLYAPLVITGVHQTTLAIDMQMVQSMGGTPVWPLIALSNIA
QASAVVGIIISSRKHNEREISVPAAISAYLGV**A**EPAMYSINIKYRFPMLCAMIGSGLAGLLCGLNGVMAN
GIGVGGLPGILSIQPTYWQVFAMAMVIAIVIPVILTTFIYQRKHRQGTLQIV

35 C (ii), *treB* A383T, GenBank: MEM6096792.1

MSKVKQADIDRLIDLVGGRDNIATVSHCITRLRFVLHQPANARPKEIEQLPMVKGCFTNAGQFQVVIGTE
VGDYYNALLETTGKAYADKEQAKKAARQNMKWHEQLISHFAEIFFPLLPALISGGLILGFRNVIGDVPMS
HGQTLAQMHPALKTLYDFLWLIGEAIFFYLPVGICWSAVKKMGGTPILGIVLGVTLVSPQLMNAYLLGQQ
TPDVWNFGVFSIEKIGYQAQVIPALLAGLALGFIETRLKRIVPDYLYLVVVPVCSLILAVFLAHAFIGPF
GRMIGDGVAFAVRYLMTGSFAPIGAALFGFLYAPLVITGVHQTTLAIDMQMVQSMGGTPVWPLIALSNIA
QASAVVGIIISSRKHNEREISVPAAISAYLGV**T**EPAMYSINIKYRFPMLCAMIGSGLAGLLCGLNGVMAN
GIGVGGLPGILSIQPTYWQVFAMAMVIAIVIPVILTTFIYQRKHRQGTLQIV

MFS transporter

30 C (i)-Ref, GenBank: MEM6145904.1

MYLREVFYYPWNEYFSLTNTQSGMLMSWLGFVGIISGAVSGIIVDRFKNPKSILTIAYLTMAALAIWQSF
RPSYQAMFIIVGFMSLVGNGLFLVSMTKIARLLASDNEQGRYFGFLESGRGIAGTVLTLCAVAIVGLHGS
SAVSIGFILRFDAAIYIILGFTSYYLFPKGVSAIENAAPKKMSDLISLLKSVKLWLAAFIISCVIFVYQG
GAYLVPYLSDAYG**M**TPDQTAVIGMIRAYFLAFIISPFAGLLADKIGSSLKVMASFFILGALITASFIFIP
HDSRFLILLITLVLLLGALTFGMRGIMYAQVNEIRIPKVFTGTAMGILICIGFSPEAYVHLIFGYLLDTY
KEFAYTLMFATIAVVLFAGALCSGLLYRINKKV

35 C (ii), M224V, GenBank: MEM6096293.1

MYLREVFYYPWNEYFSLTNTQSGMLMSWLGFVGIISGAVSGIIVDRFKNPKSILTIAYLTMAALAIWQSF
RPSYQAMFIIVGFMSLVGNGLFLVSMTKIARLLASDNEQGRYFGFLESGRGIAGTVLTLCAVAIVGLHGS
SAVSIGFILRFDAAIYIILGFTSYYLFPKGVSAIENAAPKKMSDLISLLKSVKLWLAAFIISCVIFVYQG
GAYLVPYLSDAYG**V**TPDQTAVIGMIRAYFLAFIISPFAGLLADKIGSSLKVMASFFILGALITASFIFIP
HDSRFLILLITLVLLLGALTFGMRGIMYAQVNEIRIPKVFTGTAMGILICIGFSPEAYVHLIFGYLLDTY
KEFAYTLMFATIAVVLFAGALCSGLLYRINKKV

N-acetyltransferase

30 C (i)-Ref, GenBank: MEM6145947.1

MSEIVIRHAEPKDYDAIRQIHAQPEVYHNTLQVPHPSLEMWQARLTEQAGVKQLVACIDDIVVGHLSIQV
TQ**R**PRRSHVADFGICVDARWHNRGIASALIRTMIDMCDNWLRVERIELTVFVDNEPAVAVYKKYGFEIEG
TGKKYGLRNGEYVDTYFMARVK

35 C (ii), *yhhY* R73H GenBank: MEM6096250.1,

MSEIVIRHAEPKDYDAIRQIHAQPEVYHNTLQVPHPSLEMWQARLTEQAGVKQLVACIDDIVVGHLSIQV
TQ**H**PRRSHVADFGICVDARWHNRGIASALIRTMIDMCDNWLRVERIELTVFVDNEPAVAVYKKYGFEIEG
TGKKYGLRNGEYVDTYFMARVK

pyridoxal phosphate-dependent aminotransferase

30 C (i)-Ref, GenBank: MEM6148091.1

MRNNPLIPKSKLPNLGTTIFTQMSALAQKHQAINLSQGFPDFDGPSYLHE**R**LAYHVAQGANQYAPMTGAQ
ALREAIADKTAEIYGYRPDDVSDITVTAGATEALYAAITALVRAGDEVICFDPSYDSYAPAVALSGGVLK
RIALTPPHFRVDWQAFSALLSERTRLVILNTPHNPTATVWRQADIEALWQAIGEREIYVLSDEVYEHICF
AAEGHASVLAHPQLRERAVAVSSFGKTFHMTGWKIGYCVAPAAISAEIRKVHQYLTFCVNTPAQLALADM
LRAAPEHYRALPDFYRKKRDVLVNALAQSRLKVLPCEGTYFLLIDYSAVSTLNDVEFCQWLTEEVGVAAI
PLSVFCAAPFPHQLIRLCFAKQESTLLAAAERLCKL

35 C (ii), *aspB* R51W, GenBank: MEM6098260.1

MRNNPLIPKSKLPNLGTTIFTQMSALAQKHQAINLSQGFPDFDGPSYLHE**W**LAYHVAQGANQYAPMTGAQ

ALREAIADKTAEIYGYRPDDVSDITVTAGATEALYAAITALVRAGDEVICFDPSYDSYAPAVALSGGVLK
RIALTPPHFRVDWQAFSALLSERTRLVILNTPHNPTATVWRQADIEALWQAIGEREIYVLSDEVYEHICF
AAEGHASVLAHPQLRERAVAVSSFGKTFHMTGWKIGYCVAPAAISAEIRKVHQYLTFCVNTPAQLALADM
LRAAPEHYRALPDFYRKKRDVLVNALAQSRLKVLPCEGTYFLLIDYSAVSTLNDVEFCQWLTEEVGVAAI
PLSVFCAAPFPHQLIRLCFAKQESTLLAAAERLCKL

sulfofructosephosphate aldolase

30 C (i)-Ref, GenBank: MEM6148439.1

MNNYTIKDITRASGGFAMLAVDQREAMRLMFAAAGAKTPVADSVLTDFKVNAAKILSPYASAVLLDQQFC
YRQAVEQNAVAKSCAMIVAADDFIPGNGIPVDNVVIDKKINAQAVKRDGAKALKLLVLWRSDEDAQQRLD
MVKEFNELCHSNGLLSIIEPVVRPPRCGDKFDREQAIIDAAKELGDSGADLYKVEMPLYGKGARSDLLTA
SQRLNGHINMPWVILSSGVDEKLFPRAVRVAMEAGASGFLAGRAVWSSVIGLPDTELML**R**DVSAPKLQRL
GEIVDEMMAKRR

35 C (ii), *yihT* R270C, GenBank: MEM6098419.1

MNNYTIKDITRASGGFAMLAVDQREAMRLMFAAAGAKTPVADSVLTDFKVNAAKILSPYASAVLLDQQFC
YRQAVEQNAVAKSCAMIVAADDFIPGNGIPVDNVVIDKKINAQAVKRDGAKALKLLVLWRSDEDAQQRLD
MVKEFNELCHSNGLLSIIEPVVRPPRCGDKFDREQAIIDAAKELGDSGADLYKVEMPLYGKGARSDLLTA
SQRLNGHINMPWVILSSGVDEKLFPRAVRVAMEAGASGFLAGRAVWSSVIGLPDTELML**C**DVSAPKLQRL
GEIVDEMMAKRR

undecaprenyl-phosphate galactose phosphotransferase WbaP

30 C (i)-Ref, GenBank: MEM6148439.1

MDNIDNKYNPQLCKIFLAISDLIFFNLALWLSLGCVYFTFDQVQRFIPQDQLDTRVITHFILSVVCVGWF
WIRLRHYTYRKPFWYELKEIFRTIVIFAIFDLALIAFTKWQFSRYVWVFCWTFAIILVPFFRALTKHLLN
KLGIWKKKTIILGSGQNARGAYSALQSEEMMGFDVIAFFDTDASDAEINMLPVIK**D**TGTIWDLNRTGDVH
YILAYEYTELEKTHFWLRELSKHHCRSVSVVPSFRGLPLYNTDMSFIFSHEVMLLRIQNNLAKRSSRFLK
RTFDIVCSIIILIIASPLMIYLWYKVTRDGGPAIYGHQRVGRHGKLFPCYKFRSMVMNSQEVLKELLAND
PIARAEWEKDFKLKNDPRITAVGRFIRKTSLDELPQLFNVLKGDMSLVGPRPIVSDELERYCDDVDYYLM
AKPGMTGLWQVSGRNDVDYDTRVYFDSWYVKNWTLWNDIAILFKTAKVVLRRDGAY

35 C (ii), *rfbP* D196G, GenBank: MEM6099274.1

MDNIDNKYNPQLCKIFLAISDLIFFNLALWLSLGCVYFTFDQVQRFIPQDQLDTRVITHFILSVVCVGWF
WIRLRHYTYRKPFWYELKEIFRTIVIFAIFDLALIAFTKWQFSRYVWVFCWTFAIILVPFFRALTKHLLN
KLGIWKKKTIILGSGQNARGAYSALQSEEMMGFDVIAFFDTDASDAEINMLPVIK**G**TGTIWDLNRTGDVH
YILAYEYTELEKTHFWLRELSKHHCRSVSVVPSFRGLPLYNTDMSFIFSHEVMLLRIQNNLAKRSSRFLK
RTFDIVCSIIILIIASPLMIYLWYKVTRDGGPAIYGHQRVGRHGKLFPCYKFRSMVMNSQEVLKELLAND
PIARAEWEKDFKLKNDPRITAVGRFIRKTSLDELPQLFNVLKGDMSLVGPRPIVSDELERYCDDVDYYLM
AKPGMTGLWQVSGRNDVDYDTRVYFDSWYVKNWTLWNDIAILFKTAKVVLRRDGAY

HTH-type transcriptional regulator GalR

30 C (i)-Ref, GenBank: MEM6149316.1

MATIKDVARLAGVSVATVSRVINDSPKASEASRLAVTSAMESLSYHPNANARALAQQATETLGLVVGDVS
DPFFGAMVKAVEQVAYHTGNFLLIGNG**Y**HNEQKERQAIEQLIRHRCAALVVHAKMIPDADLASLMKQIPG
MVLINRILPGLEHRCVALDDRYGAWLATRHLIQQGHTRIGYICSNHTISDAEDRLRGYYDALAESHIPAN
DRLVTFGEPDESGGEQAMTELLGRGRHFTAVACYNDSMAAGAMGVLNDNGVGVPGEVSLIGFDDVLVSRY
VRPRLTTIRYPIVTMATQAAELALALAGKCPTPEVTHVFSPTLVRRHSVSTPTDTGHLSTTD

35 C (ii), *galR* Y98H, GenBank: MEM6100309.1

MATIKDVARLAGVSVATVSRVINDSPKASEASRLAVTSAMESLSYHPNANARALAQQATETLGLVVGDVS
DPFFGAMVKAVEQVAYHTGNFLLIGNG**H**HNEQKERQAIEQLIRHRCAALVVHAKMIPDADLASLMKQIPG
MVLINRILPGLEHRCVALDDRYGAWLATRHLIQQGHTRIGYICSNHTISDAEDRLRGYYDALAESHIPAN
DRLVTFGEPDESGGEQAMTELLGRGRHFTAVACYNDSMAAGAMGVLNDNGVGVPGEVSLIGFDDVLVSRY
VRPRLTTIRYPIVTMATQAAELALALAGKCPTPEVTHVFSPTLVRRHSVSTPTDTGHLSTTD

DNA polymerase III subunit alpha

30 C (i)-Ref, GenBank: MEM6147325.1

MSEPRFVHLRVHSDYSMIDGLAKTGPLVKKAASLGMPALAITDFTNLCGLVKFYGAGHGAGIKPIVGADF
NVHNELLGDELTHLTVLAANNTGYQNLTLLISKAYQRGYGAAGPIIERDWLVELKEGLILLSGGRMGDVG
RCLLRGNQALVEECVAFYEAHFPDRYFLELIRTGRQDEETYLHAAVELAEARGLPVVATNDVRFLESDDF
DAHEIRVAIHDGFTLDDPKRPRNYSPQQYMRSEEEMCELFSDIPEALENTVEIAKRCNVTVRLGEYFLPQ
FPTGDMTTEDYLVKKSKEGLEERLAFLFPDEEERKKRRPEYDERLDIELQVINQMGFPGYFLIVMEFIQW
SKDNGVPVGPGRGSGAGSLVAYALKITDLDPLEFDLLFERFLNPERVSMPDFDVDFCMEKRDQVIEHVAD
MYGRDAVSQIITFGTMAAKAVIRDVGRVLGHPYGFVDRISKLVPPDPGMTLAKAFEAEPQLPEIYEADEE
VRALIDMARKLEGVTRNAGKHAGGVVIAPTKITDFAPLYCDEEGKHPVTQFDKSDVEYAGLVKFDFLGLR
TLTIINWALEMINKRRAKNGEPPLDIAAIPLDDKKSFDMLQRSETTAVFQLESRGMKDLIKRLQPDCFED

MIALVALFRPGPLQSGMVDNFIDRKHGREELSYPDVQWQHESLKPVLEPTYGIILYQEQVMQIAQVLSGY
TLGGADMLRRAMGKKKPEEMAKQRSVFEEGAKKNGIDGELAMKIFDLVEKFAGYGFNKSHSAAYALVSYQ
TLWLKAHYPAEFMAAVMTADMDNTEKVVGLVDECWRMGLKILPPDINSGLYHFHVNDEGEIVYGIGAIKG
VGEGPIEAIIDARNQGGYFRELFDLCARTDTKKLNRRVLEKLIMSGAFDRLGPHRAALMNSLGDALKAAD
QHAKAEAIGQADMFGVLAEEPEQIEQSYASCQPWPEQVVLDGERETLGLYLTGHPINQYLKEIERYVGGV
RLKDMHPTERGKVTTAAGLVIAARVMVTKRGNRIGICTLDDRSGRLEVMLFTDALDKYQQLLEKDRILIV
SGQVSFDDFSGGLKMTAREVMDIDEAREKYARGLAISLTDRQIDDQLLNRLRQSLEPHRSGTIPVHLYYQ
RADARARLRFGATWRVSPSDRLLNDLRGLIGSEQVELEFD

35 C (ii), *dnaE* E953G, GenBank: MEM6097413.1

MSEPRFVHLRVHSDYSMIDGLAKTGPLVKKAASLGMPALAITDFTNLCGLVKFYGAGHGAGIKPIVGADF
NVHNELLGDELTHLTVLAANNTGYQNLTLLISKAYQRGYGAAGPIIERDWLVELKEGLILLSGGRMGDVG
RCLLRGNQALVEECVAFYEAHFPDRYFLELIRTGRQDEETYLHAAVELAEARGLPVVATNDVRFLESDDF
DAHEIRVAIHDGFTLDDPKRPRNYSPQQYMRSEEEMCELFSDIPEALENTVEIAKRCNVTVRLGEYFLPQ
FPTGDMTTEDYLVKKSKEGLEERLAFLFPDEEERKKRRPEYDERLDIELQVINQMGFPGYFLIVMEFIQW
SKDNGVPVGPGRGSGAGSLVAYALKITDLDPLEFDLLFERFLNPERVSMPDFDVDFCMEKRDQVIEHVAD
MYGRDAVSQIITFGTMAAKAVIRDVGRVLGHPYGFVDRISKLVPPDPGMTLAKAFEAEPQLPEIYEADEE
VRALIDMARKLEGVTRNAGKHAGGVVIAPTKITDFAPLYCDEEGKHPVTQFDKSDVEYAGLVKFDFLGLR
TLTIINWALEMINKRRAKNGEPPLDIAAIPLDDKKSFDMLQRSETTAVFQLESRGMKDLIKRLQPDCFED
MIALVALFRPGPLQSGMVDNFIDRKHGREELSYPDVQWQHESLKPVLEPTYGIILYQEQVMQIAQVLSGY
TLGGADMLRRAMGKKKPEEMAKQRSVFEEGAKKNGIDGELAMKIFDLVEKFAGYGFNKSHSAAYALVSYQ
TLWLKAHYPAEFMAAVMTADMDNTEKVVGLVDECWRMGLKILPPDINSGLYHFHVNDEGEIVYGIGAIKG
VGEGPIEAIIDARNQGGYFRELFDLCARTDTKKLNRRVLEKLIMSGAFDRLGPHRAALMNSLGDALKAAD
QHAKAEAIGQADMFGVLAEEPEQIEQSYASCQPWPEQVVLDGGRETLGLYLTGHPINQYLKEIERYVGGV
RLKDMHPTERGKVTTAAGLVIAARVMVTKRGNRIGICTLDDRSGRLEVMLFTDALDKYQQLLEKDRILIV
SGQVSFDDFSGGLKMTAREVMDIDEAREKYARGLAISLTDRQIDDQLLNRLRQSLEPHRSGTIPVHLYYQ
RADARARLRFGATWRVSPSDRLLNDLRGLIGSEQVELEFD

<u>4-hydroxyphenylacetate permease</u>

30 C (i)-Ref, GenBank: MEM6149003.1

MSDTSSALPESPESVGSHNALSTGQQTVINKLFRRLIVFLFVLFIFSFLDRINIGFAGLTMGQDLGLSAT
MFGLATTLFYATYVIFGIPSNVMLSIVGARRWIATIMVLWGIASTATMFAVGPKSLYVLRMLVGITEAGF
LPGILLYLTYWFPAFFRARANALFMIAMPATTALGSIVSGYILSLDGIFNLHGWQWLFLLEGFPSVLLGI
MVWFYLDDTPAKAKWLTAEDKKCLQEMMDNDRLTLVQPEGAISHNAMQQRSLWREVFTPIVLMYTLAYFC
LTNTLSAISIWTPQILKSFNEGSSNITIGLLAAIPQICTVLGMIYWSRHSDKHQERKHHTALPFLFAAAG
WLLASATDRNLIQLLGIVMASTGSFSAMAIFWTTPDQSISLRARAIGIAVINATGNIGSALSPVMIGWLK
DITGSFNSGLWFVASLLVVGAAIIWLIPMKASRPRATP

37 C (iii), *hypaX* K124E, GenBank: MEM6153645.1

MSDTSSALPESPESVGSHNALSTGQQTVINKLFRRLIVFLFVLFIFSFLDRINIGFAGLTMGQDLGLSAT
MFGLATTLFYATYVIFGIPSNVMLSIVGARRWIATIMVLWGIASTATMFAVGPESLYVLRMLVGITEAGF
LPGILLYLTYWFPAFFRARANALFMIAMPATTALGSIVSGYILSLDGIFNLHGWQWLFLLEGFPSVLLGI
MVWFYLDDTPAKAKWLTAEDKKCLQEMMDNDRLTLVQPEGAISHNAMQQRSLWREVFTPIVLMYTLAYFC
LTNTLSAISIWTPQILKSFNEGSSNITIGLLAAIPQICTVLGMIYWSRHSDKHQERKHHTALPFLFAAAG
WLLASATDRNLIQLLGIVMASTGSFSAMAIFWTTPDQSISLRARAIGIAVINATGNIGSALSPVMIGWLK
DITGSFNSGLWFVASLLVVGAAIIWLIPMKASRPRATP

43 C (iv), *hypaX* K124E, GenBank: MEM6039882.1

MSDTSSALPESPESVGSHNALSTGQQTVINKLFRRLIVFLFVLFIFSFLDRINIGFAGLTMGQDLGLSAT
MFGLATTLFYATYVIFGIPSNVMLSIVGARRWIATIMVLWGIASTATMFAVGPESLYVLRMLVGITEAGF
LPGILLYLTYWFPAFFRARANALFMIAMPATTALGSIVSGYILSLDGIFNLHGWQWLFLLEGFPSVLLGI
MVWFYLDDTPAKAKWLTAEDKKCLQEMMDNDRLTLVQPEGAISHNAMQQRSLWREVFTPIVLMYTLAYFC
LTNTLSAISIWTPQILKSFNEGSSNITIGLLAAIPQICTVLGMIYWSRHSDKHQERKHHTALPFLFAAAG
WLLASATDRNLIQLLGIVMASTGSFSAMAIFWTTPDQSISLRARAIGIAVINATGNIGSALSPVMIGWLK
DITGSFNSGLWFVASLLVVGAAIIWLIPMKASRPRATP

<u>N-acetylmuramoyl-L-alanine amidase AmiA</u>

30 C (i)-Ref, GenBank: MEM6149395.1

MSTFKLLKTLTSRRQVLKTGLAALTLSGMSHAIAKEETLKTSNGHSKPKTKKTGSKRLVMLDPGHGGIDT
GAIGRNGSQEKHVVLAIAKNVRAILRNHGIDARLTRTGDTFIPLYDRVEIAHKHGADLFMSIHADGFTNP
KAAGVSVFALSNRGASSAMAKYLSERENRADEVAGKKATDRDHLLQQVLFDLVQTDTIKNSLTLGSHILK

KIKPIHKLHSRTTEQAAFVVLKSPSIPSVLVETSFITNPEEERLLGTTAFRQKIATAIANGIISYFHWFD
NQKAHTKKR

37 C (iii), *amiA* V145A, GenBank: MEM6153252.1
MSTFKLLKTLTSRRQVLKTGLAALTLSGMSHAIAKEETLKTSNGHSKPKTKKTGSKRLVMLDPGHGGIDT
GAIGRNGSQEKHVVLAIAKNVRAILRNHGIDARLTRTGDTFIPLYDRVEIAHKHGADLFMSIHADGFTNP
KAAG**A**SVFALSNRGASSAMAKYLSERENRADEVAGKKATDRDHLLQQVLFDLVQTDTIKNSLTLGSHILK
KIKPIHKLHSRTTEQAAFVVLKSPSIPSVLVETSFITNPEEERLLGTTAFRQKIATAIANGIISYFHWFD
NQKAHTKKR

43 C (iv), *amiA* V145A, GenBank: MEM6039449.1
MSTFKLLKTLTSRRQVLKTGLAALTLSGMSHAIAKEETLKTSNGHSKPKTKKTGSKRLVMLDPGHGGIDT
GAIGRNGSQEKHVVLAIAKNVRAILRNHGIDARLTRTGDTFIPLYDRVEIAHKHGADLFMSIHADGFTNP
KAAG**A**SVFALSNRGASSAMAKYLSERENRADEVAGKKATDRDHLLQQVLFDLVQTDTIKNSLTLGSHILK
KIKPIHKLHSRTTEQAAFVVLKSPSIPSVLVETSFITNPEEERLLGTTAFRQKIATAIANGIISYFHWFD
NQKAHTKKR

phage baseplate assembly protein V (LEJNAJ 10515)

30 C (i)-Ref, GenBank: MEM6147547.1
MTGVTRQIGTVSAVDADRVQARVRLPECDNLRTNWLNVLQRNTQDNKDYWLPDVGEQVEVLLDANGEDGV
ILGAVYS**D**VDKPPFSDKNVRGTKYADGAEFSYNRATHTLTVRGGIERIVVEVAADISLKGKNLDLTGVTT
INGPATLNGDLEINGSAHATGNIFADGQNSNHHSH

37 C (iii), D78G, GenBank: MEM6151651.1
MTGVTRQIGTVSAVDADRVQARVRLPECDNLRTNWLNVLQRNTQDNKDYWLPDVGEQVEVLLDANGEDGV
ILGAVYS**G**VDKPPFSDKNVRGTKYADGAEFSYNRATHTLTVRGGIERIVVEVAADISLKGKNLDLTGVTT
INGPATLNGDLEINGSAHATGNIFADGQNSNHHSH

Household D

Vi polysaccharide biosynthesis glycosyltransferase TviE

59 D (i)-Ref, GenBank: MEM6074241.1
MITQEEKLAALGKTCLTLKQEKKLAQAVALIDSELPTEALTSLAMLKKAEFLHDVNETERAYALYETLIA
QNNDEARYEYARRLYNTGLAKDAQLILKKVSNGVQKKYNNYLGKINKICDLLERLEGKAIPVGTNTCIIA
MKHAILFYRNRQPRQLPVGSFGRLALCTGSLGSGGAERQISRLAIEIARKYRQKGKIGGLKVEEPVELII
RSLTPELRQDFFLKEVLEEQVEVLEIAKITGNLFDDATIESPELRLLLSHLP**P**VCKYGIKHLVPHLCERK
LDYLSVWQDSACLMIALAALIAGVPRIQLGLRGLPPVVRKRLFKPEYEPLYQALAVVPGVDFMSNNHCVT
RHYADWLKLEAKHFQVVYNGVLPPSTEPSSEVPHKIWQQFTQKTQDADTTIGGVFRFVGDKNPFAWIDFA
ARYLQHHPATRFVLVGDGDLRAEAQKRAEQLGILERILFVGASRDVGYWLQKMNVFILFSRYEGLPNVLI
EAQMVGVPVISTPAGGSAECFIEGVSGFILDDAQTVNLDQACRYAEKLVNLWRSRTGICQQTQSFLQERF
TVEHMVGTFVKTIASQPR

76 D(iv), *tviE* P263S, GenBank: MEM6056176.1
MITQEEKLAALGKTCLTLKQEKKLAQAVALIDSELPTEALTSLAMLKKAEFLHDVNETERAYALYETLIA
QNNDEARYEYARRLYNTGLAKDAQLILKKVSNGVQKKYNNYLGKINKICDLLERLEGKAIPVGTNTCIIA
MKHAILFYRNRQPRQLPVGSFGRLALCTGSLGSGGAERQISRLAIEIARKYRQKGKIGGLKVEEPVELII
RSLTPELRQDFFLKEVLEEQVEVLEIAKITGNLFDDATIESPELRLLLSHLP**S**VCKYGIKHLVPHLCERK
LDYLSVWQDSACLMIALAALIAGVPRIQLGLRGLPPVVRKRLFKPEYEPLYQALAVVPGVDFMSNNHCVT
RHYADWLKLEAKHFQVVYNGVLPPSTEPSSEVPHKIWQQFTQKTQDADTTIGGVFRFVGDKNPFAWIDFA
ARYLQHHPATRFVLVGDGDLRAEAQKRAEQLGILERILFVGASRDVGYWLQKMNVFILFSRYEGLPNVLI
EAQMVGVPVISTPAGGSAECFIEGVSGFILDDAQTVNLDQACRYAEKLVNLWRSRTGICQQTQSFLQERF
TVEHMVGTFVKTIASQPR

75 D(v), *tviE* P263S, GenBank: MEM6043555.1
MITQEEKLAALGKTCLTLKQEKKLAQAVALIDSELPTEALTSLAMLKKAEFLHDVNETERAYALYETLIA
QNNDEARYEYARRLYNTGLAKDAQLILKKVSNGVQKKYNNYLGKINKICDLLERLEGKAIPVGTNTCIIA
MKHAILFYRNRQPRQLPVGSFGRLALCTGSLGSGGAERQISRLAIEIARKYRQKGKIGGLKVEEPVELII
RSLTPELRQDFFLKEVLEEQVEVLEIAKITGNLFDDATIESPELRLLLSHLP**S**VCKYGIKHLVPHLCERK
LDYLSVWQDSACLMIALAALIAGVPRIQLGLRGLPPVVRKRLFKPEYEPLYQALAVVPGVDFMSNNHCVT
RHYADWLKLEAKHFQVVYNGVLPPSTEPSSEVPHKIWQQFTQKTQDADTTIGGVFRFVGDKNPFAWIDFA
ARYLQHHPATRFVLVGDGDLRAEAQKRAEQLGILERILFVGASRDVGYWLQKMNVFILFSRYEGLPNVLI
EAQMVGVPVISTPAGGSAECFIEGVSGFILDDAQTVNLDQACRYAEKLVNLWRSRTGICQQTQSFLQERF

TVEHMVGTFVKTIASQPR

82 D(vi), *tviE* P263S, GenBank: MEM6047821.1
MITQEEKLAALGKTCLTLKQEKKLAQAVALIDSELPTEALTSLAMLKKAEFLHDVNETERAYALYETLIA
QNNDEARYEYARRLYNTGLAKDAQLILKKVSNGVQKKYNNYLGKINKICDLLERLEGKAIPVGTNTCIIA
MKHAILFYRNRQPRQLPVGSFGRLALCTGSLGSGGAERQISRLAIEIARKYRQKGKIGGLKVEEPVELII
RSLTPELRQDFFLKEVLEEQVEVLEIAKITGNLFDDATIESPELRLLLSHLPSVCKYGIKHLVPHLCERK
LDYLSVWQDSACLMIALAALIAGVPRIQLGLRGLPPVVRKRLFKPEYEPLYQALAVVPGVDFMSNNHCVT
RHYADWLKLEAKHFQVVYNGVLPPSTEPSSEVPHKIWQQFTQKTQDADTTIGGVFRFVGDKNPFAWIDFA
ARYLQHHPATRFVLVGDGDLRAEAQKRAEQLGILERILFVGASRDVGYWLQKMNVFILFSRYEGLPNVLI
EAQMVGVPVISTPAGGSAECFIEGVSGFILDDAQTVNLDQACRYAEKLVNLWRSRTGICQQTQSFLQERF
TVEHMVGTFVKTIASQPR

85 D(vii), *tviE* P263S, GenBank: MEM6064167.1
MITQEEKLAALGKTCLTLKQEKKLAQAVALIDSELPTEALTSLAMLKKAEFLHDVNETERAYALYETLIA
QNNDEARYEYARRLYNTGLAKDAQLILKKVSNGVQKKYNNYLGKINKICDLLERLEGKAIPVGTNTCIIA
MKHAILFYRNRQPRQLPVGSFGRLALCTGSLGSGGAERQISRLAIEIARKYRQKGKIGGLKVEEPVELII
RSLTPELRQDFFLKEVLEEQVEVLEIAKITGNLFDDATIESPELRLLLSHLPSVCKYGIKHLVPHLCERK
LDYLSVWQDSACLMIALAALIAGVPRIQLGLRGLPPVVRKRLFKPEYEPLYQALAVVPGVDFMSNNHCVT
RHYADWLKLEAKHFQVVYNGVLPPSTEPSSEVPHKIWQQFTQKTQDADTTIGGVFRFVGDKNPFAWIDFA
ARYLQHHPATRFVLVGDGDLRAEAQKRAEQLGILERILFVGASRDVGYWLQKMNVFILFSRYEGLPNVLI
EAQMVGVPVISTPAGGSAECFIEGVSGFILDDAQTVNLDQACRYAEKLVNLWRSRTGICQQTQSFLQERF
TVEHMVGTFVKTIASQPR

PTS Trehalose transporter protein

59 D (i)-Ref (GenBank: MEM6072057.1)
MSKVKQADIDRLIDLVGGRDNIATVSHCITRLRFVLHQPANARPKEIEQLPMVKGCFTNAGQFQVVIGTE
VGDYYNALLETTGKAYADKEQAKKAARQNMKWHEQLISHFAEIFFPLLPALISGGLILGFRNVIGDVPMS
HGQTLAQMHPALKTLYDFLWLIGEAIFFYLPVGICWSAVKKMGGTPILGIVLGVTLVSPQLMNAYLLGQQ
TPDVWNFGVFSIEKIGYQAQVIPALLAGLALGFIETRLKRIVPDYLYLVVVPVCSLILAVFLAHAFIGPF
GRMIGDGVAFAVRYLMTGSFAPIGAALFGFLYAPLVITGVHQTTLAIDMQMVQSMGGTPVWPLIALSNIA
QASAVVGIIISSRKHNEREISVPAAISAYLGVAEPAMYSINIKYRFPMLCAMIGSGLAGLLCGLNGVMAN
GIGVGGLPGILSIQPTYWQVFAMAMVIAIVIPVILTTFIYQRKHRQGTLQIV

60 D (ii), *treB* A383T, (GenBank: MEM6088628.1)
MSKVKQADIDRLIDLVGGRDNIATVSHCITRLRFVLHQPANARPKEIEQLPMVKGCFTNAGQFQVVIGTE
VGDYYNALLETTGKAYADKEQAKKAARQNMKWHEQLISHFAEIFFPLLPALISGGLILGFRNVIGDVPMS
HGQTLAQMHPALKTLYDFLWLIGEAIFFYLPVGICWSAVKKMGGTPILGIVLGVTLVSPQLMNAYLLGQQ
TPDVWNFGVFSIEKIGYQAQVIPALLAGLALGFIETRLKRIVPDYLYLVVVPVCSLILAVFLAHAFIGPF
GRMIGDGVAFAVRYLMTGSFAPIGAALFGFLYAPLVITGVHQTTLAIDMQMVQSMGGTPVWPLIALSNIA
QASAVVGIIISSRKHNEREISVPAAISAYLGVTEPAMYSINIKYRFPMLCAMIGSGLAGLLCGLNGVMAN
GIGVGGLPGILSIQPTYWQVFAMAMVIAIVIPVILTTFIYQRKHRQGTLQIV

75 D (v), *treB* A383T, (GenBank: MEM6045645.1)
MSKVKQADIDRLIDLVGGRDNIATVSHCITRLRFVLHQPANARPKEIEQLPMVKGCFTNAGQFQVVIGTE
VGDYYNALLETTGKAYADKEQAKKAARQNMKWHEQLISHFAEIFFPLLPALISGGLILGFRNVIGDVPMS
HGQTLAQMHPALKTLYDFLWLIGEAIFFYLPVGICWSAVKKMGGTPILGIVLGVTLVSPQLMNAYLLGQQ
TPDVWNFGVFSIEKIGYQAQVIPALLAGLALGFIETRLKRIVPDYLYLVVVPVCSLILAVFLAHAFIGPF
GRMIGDGVAFAVRYLMTGSFAPIGAALFGFLYAPLVITGVHQTTLAIDMQMVQSMGGTPVWPLIALSNIA
QASAVVGIIISSRKHNEREISVPAAISAYLGVTEPAMYSINIKYRFPMLCAMIGSGLAGLLCGLNGVMAN
GIGVGGLPGILSIQPTYWQVFAMAMVIAIVIPVILTTFIYQRKHRQGTLQIV

85 D (vii), *treB* A383T, (GenBank: MEM6061899.1)
MSKVKQADIDRLIDLVGGRDNIATVSHCITRLRFVLHQPANARPKEIEQLPMVKGCFTNAGQFQVVIGTE
VGDYYNALLETTGKAYADKEQAKKAARQNMKWHEQLISHFAEIFFPLLPALISGGLILGFRNVIGDVPMS
HGQTLAQMHPALKTLYDFLWLIGEAIFFYLPVGICWSAVKKMGGTPILGIVLGVTLVSPQLMNAYLLGQQ
TPDVWNFGVFSIEKIGYQAQVIPALLAGLALGFIETRLKRIVPDYLYLVVVPVCSLILAVFLAHAFIGPF
GRMIGDGVAFAVRYLMTGSFAPIGAALFGFLYAPLVITGVHQTTLAIDMQMVQSMGGTPVWPLIALSNIA
QASAVVGIIISSRKHNEREISVPAAISAYLGVTEPAMYSINIKYRFPMLCAMIGSGLAGLLCGLNGVMAN
GIGVGGLPGILSIQPTYWQVFAMAMVIAIVIPVILTTFIYQRKHRQGTLQIV

transposase family protein (DDE-type integrase/transposase/recombinase)

59 D (i)- Ref, GenBank: MEM6073260.1

MNAALTERLVYVARAARDAGHGKRGAIYDAACAELGMSRATLLRRLKEVSVTDKRKKRADAGRSALTRDE
AALISATLREATRKNGKRLYSIADAVETLRANGFISAGRTDETTGEFFPLSEDAISRALRNYGLHPEQLD
APAPYTEVASLHPNHVWQIDASLCTLYYLSNGHKGLQVMDSAKFYKNKPANLARIASDRVWSYEITDHAS
GWIYVEYVTGAESGENLCSVLINAMQ**E**RGGADVLHGVPKILYLDPGSANTAGMTKNMCRSLGIDLIAHKP
HNARATGQVEKARDIIERKLEPGLKFRPVHSLEELNALAAKWRSHFNATAVHSRHGKTRTDIWLKITAEQ
LKKAPSVEVCRELAVAAPELRKVTPKLRVSFRGTEFDVSTVPGVLVGEKLMITRNPWRSDVAQVVLTGED
GHETFFLVEEVRKNEFGFAEGAAVFGESYKALPETPAQMAAKETEALVTGTDNAADAAAARKAKALPFGG
RLDPYKHIDDATLPACIPKRGQASDVRGPRTEQRPMTHVEAAKALRGKFSANGLTWTPEHYRQLTAQYPD
GVPEAALDEVMATLTTPARSSVISIVNGN

77 D (iii)

E237*
