# Supplementary material for: Salmonella Typhi Haplotype 58 biofilm formation and genetic variation in isolates from typhoid fever patients with gallstones in an endemic setting in Kenya
Source: Front Cell Infect Microbiol. 2024 Nov 13;14:1468866. doi: 10.3389/fcimb.2024.1468866 (PMC11599249; doi:10.3389/fcimb.2024.1468866)
Supplement: Supplementary file 2 [file Table1.docx]

|  | **Study Participants**  **Followed-up** | **Days between index case and follow-up sample collection** | | | | | | | | | | | |
| --- | --- | --- | --- | --- | --- | --- | --- | --- | --- | --- | --- | --- | --- |
|  |  | **Month 1** | **Month 2** | **Month 3** | **Month 4** | **Month 5** | **Month 6** | **Month 7** | **Month 8** | **Month 9** | **Month 10** | **Month 11** | **Month 12** |
| HHA | *Index Case | - | **31**, **35, 39**, 60 | **88** | - | 143 | 172 | - | 214 | 241, 269 | 300 | - | 335 |
|  | Household  Contact 1 | - | **31**, 35, 39, 60 | 88 | - | 143 | - | - | - | - | - | - | - |
| HHB | Index case | 26, 29 | 33, 40, 54 | 82 | - | 138 | - | - | - | - | - | - | - |
|  | Household  Contact 1 | 26, 29 | 33, 40, 54 | 82 | - | 138 | - | - | - | - | - | - | - |
|  | *Household Contact 2 | 26, **29** | **33**, **40**, 54 | 82 | - | 138 | - | - | - | - | - | - | - |
| HHC | *Index Case | **22**, 25, 30 | 37, 51 | **78** | - | - | **170** | 203 | 239 | 268 | - | 303 | - |
|  | Household  Contact 1 | 22, 25, 30 | 37, 51 | - | - | - | - | - | - | - | - | - | - |
| HHD | *Index Case | **21**, **24**, **28** | **35** | **76** | **107** | - | - | - | - | - | - | - | - |
|  | Household  Contact 1 | 21, 24, 28 | 35 | 76 | 107 | - | - | - | - | - | - | - | - |
| HHB | Index case | 26, 29 | 33, 40, 54 | 82 | - | 138 | - | - | - | - | - | - | - |
|  | Household  Contact 1 | 26, 29 | 33, 40, 54 | 82 | - | 138 | - | - | - | - | - | - | - |
|  | *Household Contact 2 | 26, **29** | **33**, **40**, 54 | 82 | - | 138 | - | - | - | - | - | - | - |
| HH=Household  *Study participants with gallstones  Shaded cells indicate the month during which *S.* Typhi shedding was detected  The number of days between the diagnosis of the index case with typhoid fever and the detection of shedding is shown in bold  -Sample not collected | | | | | | | | | | | | | |

Supplementary Table S1: Patterns of *S*. Typhi shedding by Index cases and household contacts
